# Supplementary figures and images for: Population genomics of louping ill virus provide new insights into the evolution of tick-borne flaviviruses
Source: PLoS Negl Trop Dis. 2020 Sep 14;14(9):e0008133. doi: 10.1371/journal.pntd.0008133 (PMC7515184; doi:10.1371/journal.pntd.0008133)

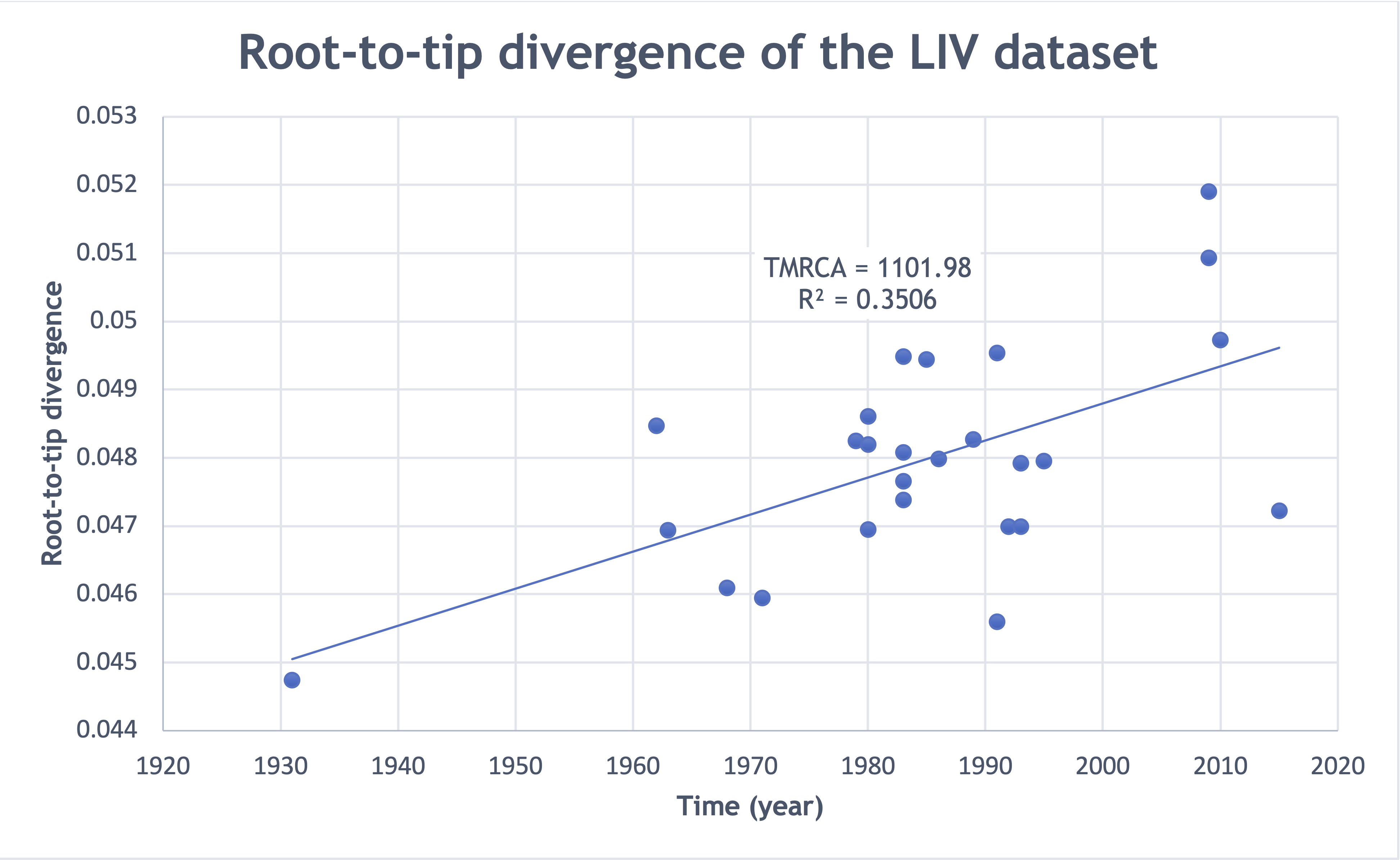

Supplement: S1 Fig — Genetic divergence was based on a ML tree generated using the dataset of 26 LIV genomes. The genetic distances from the root to the tips of the ML tree are plotted against the year of isolation. The regression line is plotted according to the best fitting root which minimises the sum of the squared residuals from the regression line. The x-intercept of the regression line represents the TMRCA, while the gradient of the line represents the clock rate. The correlation coefficient (R2) estimates the dispersion of the residuals around the regression line. The plot was generated using TempEst [56]. (TIFF) [file pntd.0008133.s001.tiff]

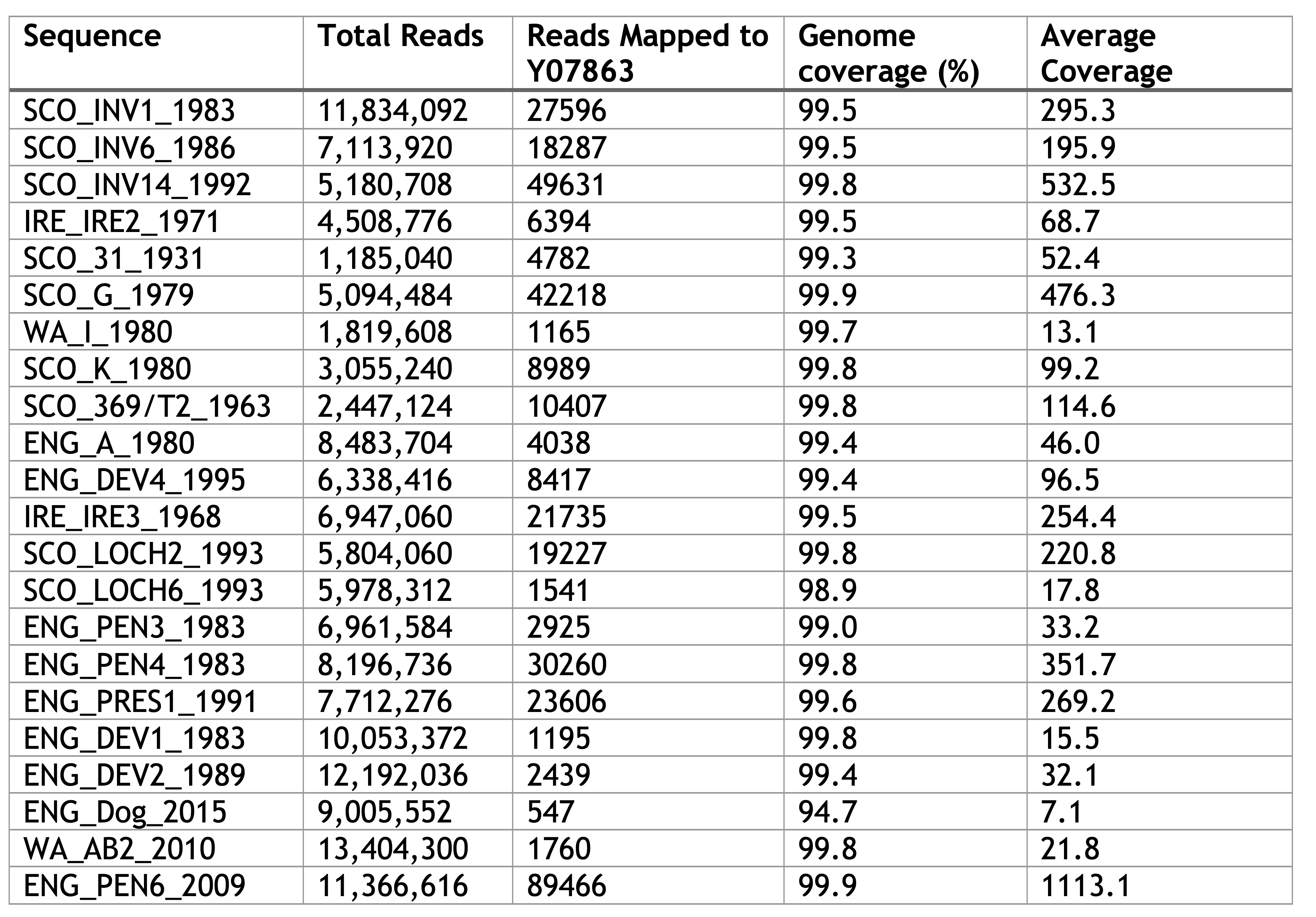

Supplement: S1 Table — (TIFF) [file pntd.0008133.s002.tiff]

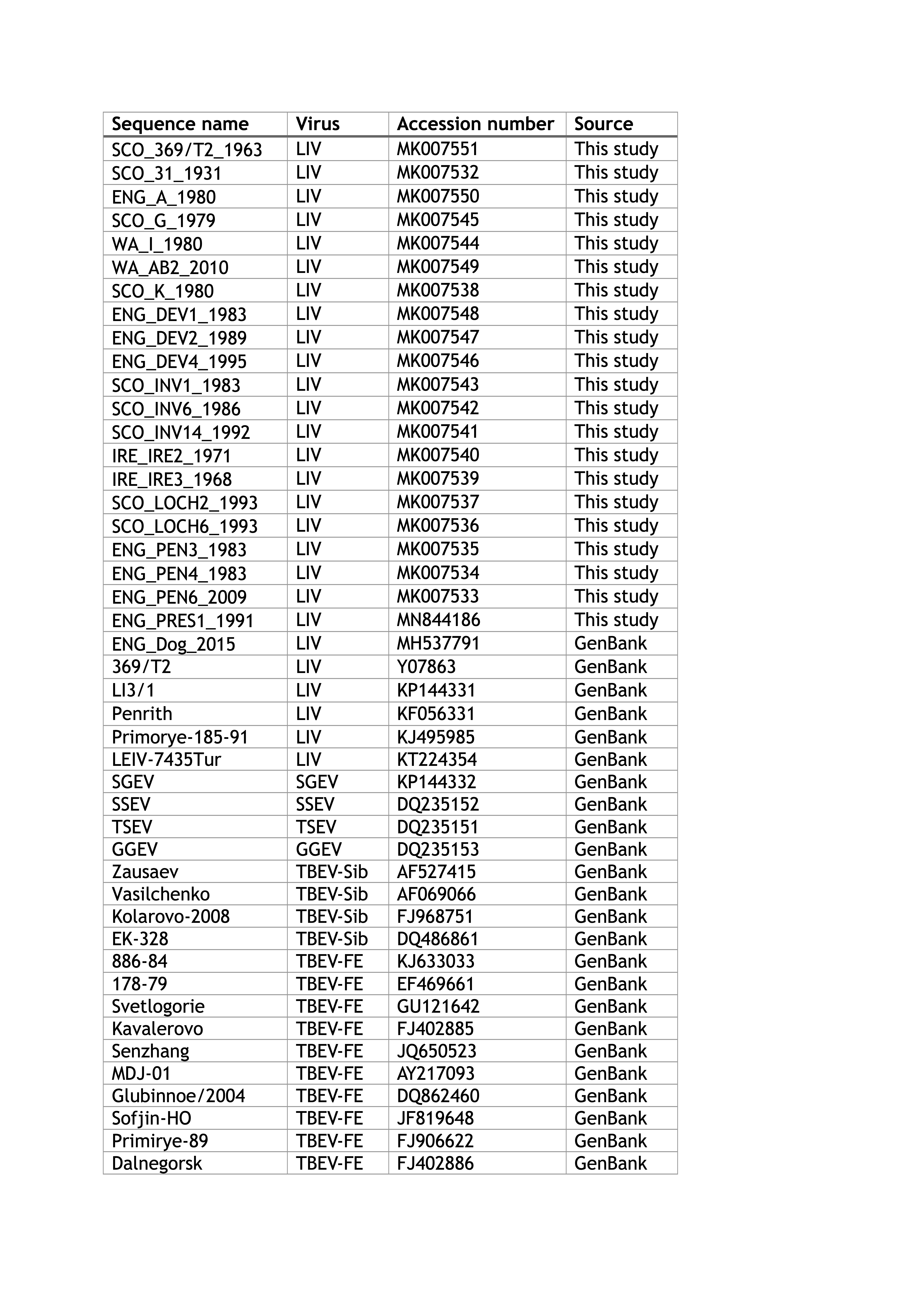

Supplement: S2 Table — (TIFF) [file pntd.0008133.s003.tiff]
