# Supplementary material for: Population genomics of louping ill virus provide new insights into the evolution of tick-borne flaviviruses
Source: PLoS Negl Trop Dis. 2020 Sep 14;14(9):e0008133. doi: 10.1371/journal.pntd.0008133 (PMC7515184; doi:10.1371/journal.pntd.0008133)
Supplement: Attachment — Submitted filename: Responses to reviewers.docx [file pntd.0008133.s004.docx]

**Responses to reviewers**

We thank reviewers for constructive comments. Our responses are outlined **in blue** below. We have left the sections as originally received as it may make it easier for reviewers to see responses to their comments, and that of other reviewers.

**Methods**

Reviewer #1: The authors have used appropriate methods

**We thank the reviewer for this comment.**

Reviewer #2: The methods are in general really well done, one minor caveat, with the selection analysis. For selection analysis you should use IFEL as well. In general, I would treat any that are only found in one method with a great deal of suspicion. Especially as they have very different P-values. Only those found in all three methods would be considered statistically significant.

**We used the Data Monkey website to perform selection analysis. While previous versions of this site included the IFEL method of selection analysis, this approach is highly similar to the fixed effects likelihood (FEL) model and appears to have been phased out from the most recent iteration of the software suite. Other methods available (FUBAR, SLAC) are not suited for small data sets like ours. We therefore were forced to restrict our analyses to the FEL model and the mixed effects model of evolution (MEME).**

Reviewer #3: Major comments:

The study design is appropriate overall but does not go deep enough into the analysis in a number of cases. The positive selection analysis is limited to the detection of statistically significant signal with very little downstream investigation, and the phylogeographic perspective on the phylogeny does not involve phylogeographic inference or any type of historic data that could corroborate the authors observations.

**Thank you for the constructive feedback. We respond to these criticisms in more detail below and we therefore describe how we addressed them at that point.**

Sequence assembly: The authors describe that they have been assembling the LIV genomes by mapping to a reference (Y07863) which they later find to be potentially inaccurate. Have they tried re-mapping to a different reference as a control? Also, when the mapped and reference sequences are divergent enough, the reference can bias mapping and consensus determination. Have the authors tried de novo assembly to control for the accuracy of the consensus they determined using reference mapping? De novo assembly can be problematic when to few reads are available but for those samples with a sufficient number of reads this could probably be done.

**While we found that the reference sequence Y07863 was potentially inaccurate, we believed that these inaccuracies were due to biological errors which arose due to the co-culture of LIV and TBEV, not through bioinformatic analysis. As the LIV genomes shared ~97% nucleotide identity, we considered it unlikely that this level of divergence would bias our assembly. Regardless, we carried out *de novo* assembly for the first 12 LIV isolates we were able to sequence. The genomes produced by both *de novo* and reference assembly methods were identical, therefore we used the reference genome for all subsequent assemblies. We have included this information in them methods section of the manuscript on lines 186-190.**

Recombination analysis: In the recombination screening section, the methods are described but there is no information on the criteria for considering a recombination event as "true". Could you please specify these criteria? For example "a p-value < 0.05 with more than 3 methods was considered as a robust recombination signal". Please also provide the results obtained for the novel sequence (e.g. a graph as a supplementary data).

**Thank you for pointing this out. We have addressed this in the methods section of the revised document on lines 204-206. As for the results obtained from the novel sequence, if no recombination event is detected within RDP4, a graph is not produced, therefore this cannot be incorporated into the supplementary data. Fig 2 displays the recombinant region for both the existing and novel LIV 369 sequences and allows for quick comparison of this region between the sequences.**

**Results**


Reviewer #1: Results are relevant and clearly presented

**We thank the reviewer for this comment.**

Reviewer #2: I have a few minor issues with the tables, in their presentation otherwise all the results are well presented.

Table 1, identities are not equal throughout the whole data set, please make clear in the legend that these are the ranges and not the same for each virus.
Table 2 – legends do not reflect the table, please correction Me is not used M is.

**Thank you for noticing this error, this has been corrected in the revised document on line 353..**

Reviewer #3: The analysis presented matches the analysis plan. However, the authors do not go deep enough into the analysis in some cases. Instigating further the sites with evidence of positive evolution or digging for historical data corroborating the authors' observations regarding the phylogeography of LIV would be most valuable for the study. On the spatial dynamics results, several of the observations made by the authors rely on a small number of sequences and are not necessary in line with the data present in the tree. Please, re-write that section more cautiously considering that you do not have a very important sampling over time and space.

**Thank you for the feedback, please see our response below.**

Major comments:

Selection analysis:

The selection analysis is limited to identifying statistically significant signal using to different methods with no further investigation. The results are fairly descriptive with no insight on possible functional evolution. For sites with statistically significant signal of positive selection it would be very valuable to go deeper into the analysis. A few examples of the questions that would be worth asking:

Are mutations a the site occurring several times in the phylogeny?
Is it only in present in recent sequences?
Is it occurring in a conserved area of the genome?
Is there literature on this specific mutation/site/region of the genome?
When you have a deep enough coverage do you have population mixture at this site?

For example, the mutation in NS3, occurs in different parts of the tree, at different
points in time, occurs in a region with low dN/dS...

**We have addressed these comments in the revised document by providing a more detailed description of where in the phylogeny these site occur, and how they may influence the protein structure and function (lines 516-529 lines 537-541, and lines 542-551). However, as LIV is poorly studied, there are essentially no published studies which have investigated the functions of the non-structural proteins. Instead, we have gleaned information from studies on TBEV and other flaviviruses. Without further studies into these functional regions our discussion is limited to conjecture. Since sequencing was done after limited passing, it would not have been clear if any sub-consensus variation would have been representative of the original sample. We therefore restricted our analysis to consensus level inference.**

Phylogeny:

Overall, I do not agree with a number of the conclusions of the authors on the phylogenetics/spatial dynamics section:

l356-357: "The tree contained repeated evidence of geographic clustering, even for samples taken decades apart."

There are several exceptions to this statement, the sequences from Ireland do not cluster together,
the sequences from southern England do not cluster all together (ENG_Dog_2015 is not clustering with the rest of the sequences A, DEV1/2/4), the sequences from northern Scotland do not all cluster together either (INV1 is separate form INV6 and INV14).

**We agree that the phylogeny contains evidence of both geographic clustering and long-distance dispersal events. We have amended the discussion to more accurately reflect both findings, instead of favouring predominantly local spread. We first discuss localised transmission events on lines 571-593, and then long distance transmission on lines 594-611.**

l359-362: "SCO_LIV_31 sampled in 1931 from the Scottish borders, which grouped with English strains sampled more recently in the borders region (Penrith, ENG_PRES1_991, ENG_PEN3_1983, ENG_PEN4_1983,ENG_PEN6_2010)."

SCO_LIV_31 is still quite distant from the English strains mentioned above.

**Agreed, as discussed above we have rewritten this section of the discussion on lines 571-593.**

l362-363: "All other Scottish strains (sampled between 1962 and 1993) formed a separate
clade"

That clade also includes sequences from the south of England (ENG_Dog_2015) and from Ireland (IRE3), which make up to a third of the clade.

**Thank you for pointing this out, we have amended the discussion to more accurately reflect the phylogenetic data** **on lines** **571-593.**

One important statement that can be made from this tree (and that is made by the authors) is that the sequences from eastern Russia and Turkmenistan probably have an origin in the UK/Ireland. This is a very interesting point to discuss in terms of historical data that may support or contradict this observation.

**We agree that the movement of LIV from the UK to Russia and Turkmenistan is very interesting, however there is very limited historical data to inform how this may have taken place. We have searched through the literature extensively but found little published information about the historical trade of livestock between the UK and Russia. In order to find out more, we contacted Janet Hartley, a professor of International History at the London School of Economics who specialises in Russian history. She advised that the Russian empire imported good quality livestock for breeding from the eighteenth century which may well have included various breeds of sheep, however she was unable to provide published proof of this, and was unwilling to speculate on whether this may have been a possible route of introduction of LIV to Russia. Without published evidence of sheep trade between the UK and Russia, and in light of our inability to accurately estimate the divergence of the Russian LIV isolates, we are hesitant to speculate further on the possible cause of the introduction of LIV into Russia. We have amended lines 594-611 to include some discussion of how and when LIV may have been transported to Russia from the UK. It would just be speculation to go further than this and we hope the reviewer appreciates this.**

Molecular clock analysis:

As it is, the order of the paragraph is surprising to me because the authors use the evolutionary rate they infer to evaluate tMRCAs for the whole species and ancestral nodes before testing (aBSREL model and randomisation) the robustness of their results.
Mentioning the tMRCA (if at all) should be done way more cautiously knowing that the evolutionary rate that was used for inference is not reliable.

**We agree that the layout of the analyses was potentially confusing and have reordered the paragraphs so that we state on lines 632-636 that our estimated clock rate is unreliable before we discuss the actual rates estimated and how they compare to other tick-borne flaviviruses on lines 638-643.**

It is not clear to me why, since you show that your estimate may not be reliable since there is an overlap with the estimates obtained using sequences with randomized dates, you repeat the analysis with the CDS without using a date-randomisation control.

**Since we failed to detect a reliable clock signal in the full data, there is no reason to suspect that working with a subset of the data would have changed that. However, we agree with the reviewer that given these concerns, there is little reason to report the results from the coding sequence only and have removed these analyses from the results.**

Minor comments:

l352-353 "increased phylogenetic certainty resulting from whole genome data"
Please illustrate your point (e.g. with a comparison) stating for which species and in which studies the split was uncertain.

**We have included a comparison of the trees we have generated with a study performed previously on lines 367-372.**


**Conclusions**

Reviewer #1: Conclusion are within bounds in relation to the results

**We thank the reviewer for this comment.**

Reviewer #2: The discussion is robust, it could have been a little more expansive on the 3'UTR which seems to be the most intriguing difference between LIV and TBEV, but that is my opinion and does not need to be changed.

**We agree with the reviewer that more information about the 3’UTR would benefit the manuscript. In dealing with a separate reviewer comment we have included some further discussion on lines 486-501** **about the 3’UTR, specifically in the context of how it modulates virulence in TBEV and how this may affect a theoretical LIV/TBEV recombinant.**

Reviewer #3: Some of the conclusion are in line with the data, others are not. The predominance of localised transmission in the dispersal dynamics is not clear based on the trees that are presented by the authors and rather suggest a mixture between localised and long-distance transmission. The authors also discuss the data based on molecular clock estimates before mentioning that their estimates are not reliable, which is misleading for the reader.

**Thank you for pointing his out, we agree that our data contains evidence of both localised and long-distance transmission and, as discussed above we have modified our discussion to reflect this.**

One of the limitations of this study is the small number of sequences with regard to the analysis. The LIV dataset is currently too small, with no longitudinal sampling to accurately describe the local dynamics of the spread of LIV.

**We agree with the reviewer and point to the need for further analyses based on larger data sets in lines 643-652.**

Specific comments:

l457-458: "Such recombination could result in novel TBFV phenotypes of potential public health concern."
Please develop here. Is there experimental or field evidence of specific lineages/species being more virulent, transmissible (or other phenotype relevant to public health) than others?

**We have expanded on this by discussing the differences in virulence observed between the TBEV subtypes, how this can be influenced by UTR length, and how recombination may result in novel phenotypes on lines 486-501.**

"Our findings could support the development, if necessary, of tools for differential diagnosis and vaccine design."
Same here, please develop on how and why. For example, you could discuss the utility of the information on diversity and purifying selection analysis for vaccine and drug development.

**We have expanded on this by further discussing how our dataset could be used to generate LIV and TBEV specific qPCRs to aid differential diagnosis between these closely related viruses. This is discussed on lines 565-570. We also discuss how our positive selection analysis may aid vaccine design on lines 546-551.**

l472-473: "Codon site 100 within NS2A was identified as being under positive selection as was codon 96 within NS3."
l480-481:"Sites 522 and 699 within NS5 were also identified as being subjected to positive selection."
Here you're simply stating your results, please develop on this statements or remove.

**Please see the response below.**

l479-480: "SGEV exhibits a histidine at codon 434, further indicating that histidine is the ancestral codon."
Please develop on the possible functional consequences of histidine being the ancestral codon, otherwise the relevance of this information is not clear.

**We have expanded on the above points by referencing functional studies from other flaviviruses which detail the roles carried out by the NS proteins, and how the observed positive selection may influence these on lines 516-529 lines 537-541, and lines 542-551.**

l496-498:"Bayesian phylogenetic analysis of the LIV dataset revealed that the Irish and Welsh isolates tended to cluster separately from the English and Scottish isolates, with the exception of IRE_IRE3_1968."
Since there are only two Irish sequences, only the Welsh isolates cluster separately then.

**Thank you for this input. We have re-written this section of the discussion with the reviewer’s points taken into consideration, as discussed above.**

l496-519: I don't agree with most of the paragraph, there is possibly a degree of local transmission but there are also movements between Ireland and the UK, North and South and given the current sampling and data it is not possible conclude that one type of transmission is predominant over the other.

On the introduction from western Scotland to Ireland please be more cautious since there are only three sequences sampled several years apart.

**We agree with the assessment that the tree contains evidence of both local and long-range transmission and have made amendments to our discussion to present a more balanced treatment of this issue as discussed above. We discuss the movement of LIV between Ireland and Western Scotland with more caution on lines 579-587.**

"from scotland to england"
Here, there is no basis for assuming any directionality in the movements between England and Scotland with certainty.

**We agree and have rectified this statement on lines 592-593.**
line 520-529:
Here it would be very insightful to have historical data on exchanges between the UK and Eastern Russia/Turkmenistan to further inform that part of the tree and possibly clarify the timing of the introduction.

**We agree with the reviewer however, as discussed above, we were unable to find good written evidence of historical livestock trade between the UK and Russia. As we cannot date the divergence time of the Russian and Turkmenistan isolates with current data, we can only theorise about the emergence of LIV in these distant countries. Using available data we provide a rough estimation of when LIV may have been introduced to Russia on lines 594-611.**

l530-535: Here I do not agree with the structure of the paragraph. The authors should start by making clear that they identified that the estimates are not reliable and then discuss the results in order to identify how the estimates could be improved or corroborated.

**We agree and have rectified this on lines 632-636 so that we first discuss that the clock rate is unreliable before we expand on how this clock rate compares to other flaviviruses on lines 638-641, before discussing how this could be improved on lines 643-652.**

l574-576:"This apparent variation between the replication rate within vertebrate host and vector species may contribute to the weak temporal signal present in the LIV and TBEV-Eu datasets"
Yes, and please discuss the other limitations that could explain the lack of temporal signal here.

**We have discussed on lines 643-652 how the limited number of sequences may have hindered our clock analysis, and how we would improve upon this.**

l581: "recombination has not contributed to LIV evolution"
You could be more cautious here because this is true, given the currently sampled viral diversity.

**We agree and have amended this on lines 685-687.**

582: "geographic isolation of LIV" I'm not so sure about that.

**As LIV is predominantly found in the UK and has likely only recently emerged in Norway, Denmark, and Russia, in comparison to TBEV it is geographically isolated. Therefore, it is likely that LIV and TBEV have only began co-circulating recently whereas previously their geographic isolation would have prevented such an event.**

590: "predominantly by localised transmission events with few long-distance
590 dispersals." Again, I do not agree with this conclusion.

**We agree that the phylogeny contains equal evidence of both localised and long-distance transmission and have amended this on lines 695-699 in order to present this more fairly.**


**Editorial and Data Presentation Modifications?**


Reviewer #2: Line 303, need to amend as this is the wrong tense
I would also not focus as much on the recombination, I think allowing this idea to fade away would be a really good thing, and so I would not focus on it other than to mention that you have eliminated the possibility due to previous sequencing errors.

**We thank the reviewer for noticing this error and have amended the tense used throughout the legend of figure 2 on lines 317-333.**

Reviewer #3: In the virus isolates section, I do not seem to find any description for isolation/sampling of strains ENG_PEN6_2009 and WA_AB2_2010.

**We thank the reviewer for noticing this oversight and have included this information on lines 146-149.**

Please introduce abbreviations (e.g. TBEV-Eu, TBEV-Sib, TBEV-FE)

**These are introduced on lines 72-73.**

l288-291: "The genetic diversity present within the LIV dataset is similar to that found for TBEV-Eu, with the exception of the 3’ UTR which is approximately 96% identical between the LIV isolates, but is highly variable between TBEV-Eu isolates (Table 1)."
Please provide the %id for the 3'UTR region in the TBEV-Eu group to illustrate your point and ease reading.

**We have included this information on lines 308-309.**

Some dates are not matching between Fig 1, and the trees. Is WA_AB2 from 2009 (tree-Fig5) or 2010 (table-Fig1)?

**We have amended the figures to display the correct date.**

Please provide access to raw sequencing data.

**We have uploaded the raw sequencing data to the European nucleotide archive, accession number PRJEB38554. We have included this information in the manuscript on lines 174-176.**

**Summary and General Comments**


Reviewer #1: Given that it seems to be fairly geographically restricted (although it is found at very geographically different locations), it would be very interesting to hear the authors ideas as for what is needed for LIV to become established in a particular area? Why is it relatively prevalent in the UK but (seemingly) not elsewhere? What habitat and ecological circumstances are necessary for LIV to become established in one area? Is it simply likely to be undersamples elsewhere?

Although the focus is clearly the evolutionary history of LIV/TBFV, I think a paragraph or so regarding the tick-virus-reservoir host-ecology relationship would complement the manuscript quite well.

**We thank the reviewer for their comments and agree that some discussion of LIV ecology would enhance the manuscript. We have added a section at lines 612-629 which describes the hosts that contribute to LIV transmission in the UK, where in the world the hosts are found, and how LIV may be under sampled elsewhere.**

Minor comments:
-r71: "...resulting in febrile illness and often fatal encephalitis (4)..."

"Often" is relative of course. But I would say that it makes more sense to mention e.g. incidence / case fatality rate.

**We have included case fatality rates for the TBEV subtypes on lines 75-77.**

-r141: "..).RNA.." Add space.

**The text has been amended on line 149.**

-r153-160: I would probably use e.g. "sequence libraries" or similar instead of "samples" to distinguish between RNA samples and libraries.

**The text has been amended on lines 150, 162, 164, 165, and 167.**

-r214: "suite(37,52)" add space

**The text has been amended on lines 227.**

-r283 and more: Check usage of range. "92.1% – 99.9%" vs. "92.1%–99.9%"

**This has been amended on lines 301-302.**

Reviewer #2: This paper is a discussion of the evolution of LIV and highlights the similarities and differences from TBEV.
The paper is coherent and has some excellent points. I have a few minor comments that I feel need to be addressed but the paper has been well designed and the analyses are in general robust.

**We thank the reviewer for their assessment and hope that the necessary changes have been made to the document.**

Reviewer #3: In this study the authors sequence complete genomes from a set of 22 Louping ill virus isolates and use them to (re)inspect evolutionary aspects such as recombination, positive selection and evolutionary rates at the level of the species.

Using RDP, they identify no evidence of recombination within the LIV species and find additional support for the absence of recombination between the isolate LIV 369/T2_Y07863 and sequences from the TBEV Eu group. Using two different maximum likelihood approaches they find evidence of positive selection within the LIV species at seven sites and show broad purifying selection with low dN/dS
ratios observed in most regions of the genome. The authors use a combination of ML and
Bayesian tree-building methods to infer the phylogenetic relationships between LIV isolates and between LIV species and the rest of the TBFV group. They use these trees, in combination with space and time information attached to the sequences to identify both localised and long-distance transmission dynamics of LIV in the UK and Ireland. Finally, using Bayesian inference they infer the evolutionary rate within the species and identify that their estimate is not reliable, using a control dataset with randomised dates.

This study is robust in terms of methodology but has several limitations in terms of analysis. The selection analysis dos not go deep enough so that this part of the results is only descriptive with no insight into possible functional evolution within LIV. The conclusions of the authors on the spatial spread of the virus do not reflect what is shown in the trees, identifying a predominantly local transmission while the trees rather point at a mixture of local and long-distance transmission with no dynamic being clearly predominant. Also, given the sampling, it is difficult to confirm local transmission, longitudinal sampling would be ideal here. Finally, the authors should make clear in their results and in the discussion section that the rate estimates they discuss are not reliable. In the current form, they start by discussing the results before mentioning that the evolutionary rates are not robust.

**We thank the reviewer for their thorough assessment of the manuscript. We agree with the points made and have taken steps to address the above concerns. We have expanded our discussion of the selection analysis, modified the discussion of our phylogenetic analysis and included as much information as is available to our discussion of the transmission of LIV to Russia.**
