# Supplementary material for: Population genomics of louping ill virus provide new insights into the evolution of tick-borne flaviviruses
Source: PLoS Negl Trop Dis. 2020 Sep 14;14(9):e0008133. doi: 10.1371/journal.pntd.0008133 (PMC7515184; doi:10.1371/journal.pntd.0008133)
Supplement: Attachment — Submitted filename: Response to reviewers 2.docx [file pntd.0008133.s005.docx]

**Responses to reviewers**

We thank reviewers for constructive comments. Our responses are outlined **in blue** below. We have left the sections as originally received as it may make it easier for reviewers to see responses to their comments, and that of other reviewers.

**Results**


Reviewer #1: The results and related figures/tables are clearly presented and match the aim of the manuscript.

**We thank the reviewer for this supportive comment.**

Reviewer #2: All my comments were adequately addressed.

**We are glad changes/modifications are sufficient.**

Reviewer #3: The authors answered all the comments regarding the results section and in most cases, corrected the manuscript wherever it was necessary. I still have some minor comments on that section:

line 350: "MEME denotes sites which have been identified by MEME"
Please correct the legend so it correctly describes what is in the table. In my understanding it should be MEME rather than ME.

**We apologise for this oversight. This has been amended on line 374.**

On the Selection analysis:

The authors provide a better description of the mutations identified in the selection analysis. I would recommend moving some of the information to the results section (e.g. which strains carry which amino acid at a given position) and in the discussion, have a documented and well-argued paragraph on why the readers should care (or not) about some of the mutations. For now that part of the discussion looks a little too much like a catalogue.

**We have moved the description of the positively selected sites to the results section on lines 334-360. We discuss one of the positively selected sites in the E gene on lines 533-542. Due to the lack of existing data on the functions of the LIV proteins, discussion of the other positively selected sites would be limited to speculation therefore we have refrained from commenting on these further.**

On the Phylogenetic analysis:

The authors have improved their conclusions on the spatial dynamics of LIV but some inaccuracies remain:

line 573-575: "This is particularly evident for the isolates collected in northern England, northern Scotland, Wales and Southern England."

As mentioned in my previous comment, not all sequences from Northern Scotland cluster together (INV1 is separate form INV6 and INV14). The authors should specify this point in the narrative, as they do for the isolate ENG_DOG_2015.

**We agree and have modified the manuscript on lines 630-634.**

General comment on paragraph 571-593:

While discussing mechanisms of localised transmission, rather than just considering small distance movements, the authors should also discuss possible mechanisms for persistence in a population over the years because this would also be an important part of the phenomenon.

**We agree that the mechanisms underlying LIV persistence should be discussed in addition to localised transmission. We have moved our discussion of this specific point to lines 601-617.**

lines 591-608: On strains from Eastern Russia and Turkmenistan:

I completely agree with the authors, if there is no or very little historical data, the authors should not speculate too much. However they can cautiously lay out several possible scenarios specifying that they are only hypotheses.

**We agree that hypothesising about the spread of LIV from the UK to Russia would improve the discussion and have discussed the possibility of migratory birds transporting infected ticks on lines 653-666**

line 594: Recent is very relative, I recommend avoiding using such qualifier, or specifying in which context it can be considered "recent".

**We agree and have modified the manuscript on lines 637-639**

lines 606-608: The last sentence is a little too speculative. I recommend making a general statement stating that based on the (scarce) historical data available, introduction of LIV into these regions might have occurred via trade routes. The authors should avoid specifying a point of entry, because there is no proof for that. Important point, the tree suggests that the lineages in Eastern Russia and Turkmenistan stemmed from the UK region, pinning down the geographical origin of the introduction is not possible given the sampling and the approach used here.

**Our attempts to hypothesise about the introduction of LIV to the Russian Far East using historical information such as the opening of Vladivostok port and the trans-Siberian railway were more an attempt at hypothesising a timeframe in which LIV may have been introduced, as opposed to a geographical origin. To reflect this we have modified lines 648- 652 to make it more clear that we are describing trade between the UK and the area of Primorsky Krai, as opposed to Vladivostok specifically.**

On the minor comment:

line 365-370: The amended version is better but I don't think this is about resolution. For this analysis, genomic sequences were more informative than single gene sequences and yielded more robust results.

**We used the term ‘resolution’ to reflect higher phylogenetic certainty and have amended line 393 to reflect this.**

Table 2 legend line 350: "ME denotes sites which have been identified by MEME"

Based on what is shown in the table, it should be MEME

**We apologise for this oversight and have amended line 374.**

--------------------

**Conclusions**

Reviewer #1: Conclusions are within bounds of what can be inferred from the data and addressed properly.

**We thank the reviewer for this comment.**

Reviewer #2: All my comments were adequately addressed

**We are glad the reviewer feels their comments have been addressed.**

Reviewer #3: The authors answered all the comments regarding the discussion section and in most cases, corrected the manuscript wherever it was necessary. I still have some minor comments on that section:

On paragraph 484-499:

When writing that there are no reverse genetics systems available for LIV the authors could however mention that reverse genetics systems developped for TBEV could most likely be harnessed for studying the phenotype of LIV/TBEV recombinants.

**We have modified the manuscript on lines 520-523 to mention the use of existing TBEV reverse genetics systems to investigate LIV/TBEV recombinants.**

lines 514-516: "the clade of isolates sampled from ... encode a serine"

In my understanding it should be "encodes".

**We thank the reviewer for noticing this and have amended the tense on line 342.**

line 581 in original manuscript: "recombination has not contributed to LIV evolution" and lines 685-687 in the amended version.

The amended version is not clear enough on that point. I suggest replacing "we have confirmed" with a more cautious formulation "we did not find evidence of recombination contributing to LIV evolution"

**We agree and thank the reviewer for their suggestion. This has been amended on lines 705-706 and on lines 498-499.**

On the comment of the authors on the "geographic isolation of LIV".

I get the point however, there is evidence that LIV is present outside the UK and might have been for some time, as indicated by the identification of LIV in samples from Eastern Russia and Turkmenistan.

**We have revised the Discussion to reflect the existence of LIV foci in other countries on lines 706-710.**

--------------------

**Editorial and Data Presentation Modifications?**

Reviewer #1: (No Response)

Reviewer #2: No changes required.

Reviewer #3: The authors corrected the manuscript wherever it was necessary. On last edit I recommend is increasing the resolution of the figures.

--------------------

**Summary and General Comments**

Reviewer #1: The authors have addressed all my previous comments. I have nothing further to add.

Reviewer #2: No changes required

Reviewer #3: The authors answered all the comments from reviewers #1, #2, and #3 and made most of the corrections that were recommended. The manuscript as greatly improved so that only a few minor modifications are now necessary for it to be ready for publication.

**We thank all of the reviewers for their important insights and hope that the necessary changes have now been made to the manuscript.**
